# Outcomes of root canal treatment of first permanent molars among children in Jeddah, Saudi Arabia: A retrospective cohort study

**DOI:** 10.1016/j.heliyon.2022.e11104

**Published:** 2022-10-15

**Authors:** Wala Dhafar, Heba Jafar Sabbagh, Abdullah Albassam, Jihan Turkistani, Rzan Zaatari, Manal Almalik, Amal Dafar, Sanaa Alhamed, Ahlam Bahkali, Nada Bamashmous

**Affiliations:** a University Dental Hospital, King Abdulaziz University, Jeddah, Saudi Arabia; b Pediatric Dentistry Department, Faculty of Dentistry, King Abdulaziz University, Jeddah, Saudi Arabia; c Department of Endodontics, Faculty of Dentistry, King Abdulaziz University, Jeddah, Saudi Arabia; d National Guard Health Affairs, King Abdulaziz Medical City, Dental Services Department, Jeddah, Saudi Arabia; e Alarak Almutamayzah Medical Company, Jeddah, Saudi Arabia; f Dental Department, King Fahad Armed Forces Hospital, Jeddah, Saudi Arabia; g Department of Oral and Maxillofacial Surgery, King Fahad General Hospital, Jeddah, Saudi Arabia; h Oral Diagnostic Sciences Department, Faculty of Dentistry, King Abdulaziz University, Jeddah, Saudi Arabia

**Keywords:** Endodontics, Root canal treatment, Success, Failure, First permanent molar

## Abstract

**Objectives:**

The first permanent molar (FPM) is considered the tooth most susceptible to caries, as it is the first permanent tooth to erupt in the oral cavity, making it susceptible to environmental conditions that may appear as caries, hypoplasia, or hypomineralization*.* Several treatment options are available for managing deep caries, including root canal treatment (RCT). However, there is a lack of data on the success and failure rates of RCT in FPM among children. This study aimed to determine the success and failure rates of RCT in FPM among children and related factors.

**Methods:**

This retrospective cohort study was conducted at three major centers in Jeddah, Saudi Arabia. Children aged 9–18 years who underwent an RCT between 2010 and 2019 were included. Clinical and radiographic examinations were also performed.

**Results:**

Based on the loose criteria, most of the evaluated teeth (79.6%) were successfully treated. The treatment failed in only 20.4% of participants. Older patients and teeth with acceptable restoration quality had an increased success rate compared to younger patients and teeth with unacceptable restoration quality. A shorter time lapse between treatment and assessment resulted in a lower success rate compared to a longer time lapse. Based on strict criteria, 72.9% of the patients were successfully treated. The use of a microscope and teeth with acceptable restoration quality resulted in an increased success rate compared to teeth treated without the microscope and with unacceptable restoration quality.

**Conclusions:**

The success rate of this procedure was high. Several factors, including older age, acceptable restoration quality, and the use of a microscope, increase the probability of success.

## 1 Introduction

Dental caries is one of the most common oral diseases in children [1]. The first permanent molar (FPM) is the tooth most susceptible to dental caries in the permanent dentition [2], as it erupts early in the oral cavity [3, 4] by 6 years of age [5]. Management of deep dental caries reaching the pulp, causing pulp inflammation and necrosis in FPM with mature roots, is achieved by root canal treatment (RCT) [6].

Several studies have been conducted globally to evaluate RCT outcomes. However, these studies mainly addressed the success and failure rates among adult patients or grouped adults with adolescents. Nevertheless, the literature lacks information on the success rate of RCT performed on the permanent first molars in children. Lazarski et al. [7] conducted a retrospective study to evaluate unwanted events, such as retreatment, apical surgery, or extraction, following nonsurgical RCT. During the five-year-period, 586,000 patients aged 14–90 years were included. The incidence of unwanted event was reported as 6.40%. In addition, a total of 94.44% of the nonsurgical RCT were functional at follow up period of 3.5 years [7]. Another study was conducted by Salehrabi and Rotstein [8] to retrospectively analyze the outcomes of initial RCT, as well as tooth retention over a period of 8 years between 1995 and 2002, without specifying the age group of the included patients. By the end of the evaluative period, 96.89% of molar teeth were retained, and only 3.11% were extracted [8].

However, managing children during RCT treatment, especially uncooperative patients is a challenge which could affect the quality of restoration, obturation in addition to treatment [9]. Furthermore, it is not possible to perform a final coronal restoration, including metal/fabricated crowns, in children [10]. These factors could affect the outcome of RCT success in young age groups. Therefore, it is essential to address the success rate of RCT treatment in younger age groups [11].

One study that was conducted on children aged 8–18 years aimed to evaluate the quality of RCT performed for pediatric patients [12]. Periapical radiographs of the 100 root-canal (RC)-treated teeth in the hospital were evaluated, and it was concluded that 61% had satisfactory RCT, while 39% had unsatisfactory RCT. The majority of unsatisfactory RCT had non-homogenous filling material, followed by extruded material and short filling [12].

Therefore, this study aimed to evaluate the RCT success and failure rates in FPM among children aging 9–18 years and related factors. The null hypothesis of the current study states that there is no difference between the success rate of RCT according to the radiographic quality of RCT at treatment time, patients' socio-demographic characteristics, dental characteristics, and clinical findings.

## 2 Materials and methods

This study was conducted at three major centers in Jeddah, Saudi Arabia: University King Abdulaziz University Hospital (KAUFD), King Fahad Armed Forces Hospital (KFAFH), and King Abdulaziz Medical Center (KAMC). Ethical approval was obtained from the Research Ethics Committee of KAUFD (172-11-19), Research Ethics Committee of KFAFH (H-01-R-005), and the Institutional Review Board of the Ministry of National Guard at KAIMRC (REC 407). The study was a retrospective cohort study with five different times, ranging from 6 months to 94 months, and exposure to different root canal treatment qualities.

This study included patients from the three aforementioned centers, at which their inclusion criteria were: (1) healthy patients, (2) aged between 9 and 18 years old at treatment time, (3) underwent RCT in FPM, (4) closed root apices (5) in the time period from 1st of September 2010 to 30th of June 2019, (6) available radiographs for treated teeth after completion of RCT, and (7) undergone treatment at least 6 months prior to assessment appointment. The six months' period was selected based on a previous study that reported a minimum of 6 months before any periapical healing occurred [13]. Moreover, in a study by Huumonen et al., no significant difference in apical periodontitis healing was found between the 3-month and 12-month follow-ups. They concluded that the 3-month follow-up period was adequate [14].

To evaluate the RCT outcomes, the list of patients aged 9–18 years was primarily filtered to exclude patients who did not fulfill the inclusion criteria (134 patients). These patients were either medically compromised, had missing radiographs, or had undergone extraction of the treated tooth. The distribution of the sample according to sex was similar in the excluded and included subjects (36.5% and 38.7% males respectively). Participants were identified and their parents were contacted via phone and invited to participate in the study. Parents who answered and agreed to their children to be included were then booked for assessment appointments for clinical and radiographic examinations. Written informed consent was signed by patients (older than 18 years) or their parents (patients younger than 18 years), and assent was obtained from the child after explaining the research purpose. Clinical and radiographic examinations were conducted by three clinicians: WD at UDH, RZ at KFAFH, and JT at KAMC. The data collection form consisted of two sections. The first section of the data collection sheet comprised the patients' general and demographic data, including file number, age, gender, nationality, family income, and parental education level. The second section included information on success and failure. The first part of this section contained information about the healthcare providers who performed the RCT. Next, information regarding the treatment system used, which can be conventional or rotary, followed by information concerning whether the microscope was used, and finally, information regarding the obturation technique. The second part of the section focused on clinical examination data. Examination at the time of RCT was recorded from the patients' files, and at the time of assessment, a clinical examination was performed. It included information about pain, pain on percussion, pain on palpation, presence of sinus tract, presence of swelling, pocket depth, tooth mobility, and type and quality of coronal restoration. The third part of the second section concentrated on radiographic examination of the previously obtained radiograph on the treatment visit, as well as the follow-up radiograph on the assessment visit. This was performed by examining the presence of the lamina dura, periodontal ligament width, periapical area and apical structures, signs of root resorption, quality of root filling material, and radiographic quality of coronal restoration. Radiographs were prescribed for assessment in accordance with the American Academy of Pediatric Dentistry (AAPD) guidelines [15], which recommend radiographic examination every 6–12 months. All patients were offered comprehensive treatment if needed.

The expected outcomes of RCT were defined as success or failure. Success was subdivided into success with strict criteria and that with loose criteria. The current study adopted the criteria of Ng et al. to determine the outcomes [16]. Treatment was considered successful based on loose criteria when there was absence of pain, absence of clinical evidence of inflammation or swelling, and radiographs showing complete healing and normal periodontal ligament space, or when there was a reduction in the size of the lesion without returning to normal periodontal ligament space width. Treatment was considered failed if a tooth was extracted or presented with pain, inflammation, swelling, sinus tract, or newly emerged or increased in size periapical radiolucent lesions [16].

### 2.1 Ascertainment

Three examiners met for the calibration and training. The data collection form was printed and reviewed by three examiners to improve the clarity and understanding of the material. The coding of each item in the data collection form was explained, discussed, and agreed upon by examiners. The inter-examiner reliability test was performed separately for clinical and radiographic examinations of patients. Their data was entered in SPSS and the result of Kappa test was 0.8 for WD and RZ, and 1 for WD and JT.

### 2.2 Content validity and reliability of the questionnaire

A panel of experts (four pediatric dentistry consultants and three endodontic consultants) assessed and evaluated the content validity of the questionnaire. Assessment of relevance, clarity, simplicity, and ambiguity for each question was performed by placing a scale from 1 to 4, with 1 being the lowest and 4 being the highest. The Content Validity Index was 0.93, indicating that the questionnaire was valid. Reliability was determined by testing internal consistency, and the Cronbach’s alpha was 0.885.

### 2.3 Sample size calculation

Sample size calculation was performed using G-power 3.1.9.7, according to Gillen et al. [17], who conducted a systematic review on the effect of quality of restoration on the success of RCT in adults. An estimated sample size of 200 was calculated using an odds ratio of 2, α error probability of 0.05, and 80% power.

### 2.4 Statistical analysis

Statistical analysis was performed using Statistical Package for the Social Sciences (SPSS) ver. 22 (IBM Corp., Armonk, NY, USA) for Mac OSX software. The threshold for statistical significance was set at *P* ≤ 0.05. The statistical analysis tests included descriptive statistics and frequencies for the qualitative data. Binary logistic regression analysis was conducted on significant variables at the time of treatment (as an independent factor) to overcome the effect of confounding factors on success based on loose and strict criteria (as dependent factors).

## 3 Results

During the study period, 204 patients were recruited, of which 52% were from KFAFH. The mean age of the patients was 14.1 years at the time, and 18.5 years at the time of assessment. Female patients accounted for 61.3% of the study population. Regarding sociodemographic factors, 77.9% of the patients came from families with moderate income, 69.6% had fathers with high education levels, and 32.4% had mothers with high education levels. Demographic data of the participants are presented in Table 1.

**Table 1:** Demographic data (n = 204).

Demographic Data		No. of Subjects (%)
Age (years)	At treatment visit mean +SD, range	14.1 ± 2.3, 9-18
	At assessment visit ​ mean +SD, range	18.5 ± 3.3, 9.5–27
Healthcare Center	UDH	58 (28.4%)
	KFAFH	106 (52%)
	KAMC	40 (19.6%)
Gender	Males	79 (38.7%)
	Females	125 (61.3%)
Nationality	Saudi	176 (86.3%)
	Non-Saudi	28 (13.7%)
Family income	Low	26 (12.7%)
	Moderate	159 (77.9%)
	High	19 (9.3%)
Father’s education level	Low	13 (6.4%)
	Moderate	49 (24%)
	High	142 (69.6%)
Mother’s education level	Low	69 (33.8%)
	Moderate	69 (33.8%)
	High	66 (32.4%)

No.: Number.

SD: Standard deviation.

RCT: Root Canal Treatment.

Among the included sample of patients, 284 FPM teeth had an RCT, of which 94 (33%) were located in the maxillary arch and 190 (66.9%) in the mandibular arch. The total number of evaluated teeth was 243 (85.6%), and the remaining 41 (14.4%) were extracted for one of the following reasons: pain, grade 3 mobility, and non-restorability. The time elapsed between treatment and assessment visits ranged from 6 months to 7.8 years and with a mean of 3.3 ± 2 years (Table 2).

**Table 2:** Characteristics of root canal treated teeth (n = 284).

Characteristics of Root Canal Treated Teeth	No. of Teeth (%)
*Location*	
Maxillary molars (16,26)	94 (33.1)
Mandibular molars (36,46)	190 (66.9)
*Presence/absence of tooth*	
Present	243 (85.6%)
Extracted	41 (14.4%)
*Reasons for extraction*	
Bifurcation involvement and grade iii mobility	1 (2.4%)
Non-restorable tooth	25 (61%)
Pain	15 (36.6%)

No.: Number.

When clinical and radiographic assessments were conducted, 226 teeth were found to have successful treatment based on loose criteria representing a success rate of 79.6%, 207 teeth were found to have successful RCT based on strict criteria representing a success rate of 72.9%, and only 58 teeth failed, representing a failure rate of 20.4%.

***Based on loose criteria***, binary logistic regression analysis was conducted, which revealed a statistically significant (*P* ≤ 0.05) relationship between success and patient age at treatment time, time lapse between treatment and assessment time, and radiographic quality of coronal restoration at the time of treatment. Patients older than 14.4 years at the time of treatment had a significantly increased probability of success compared to younger patients (*P* = 0.036, OR = 0.981, 95% CI:0.963–0.999). A shorter time lapse between treatment time and assessment time resulted in statistically significant lower probability of success compared to a longer time lapse (*P* = 0.043, OR = 4.558 and 95% CI:1.049 to 19.806). Teeth with acceptable restoration quality had a statistically significant increase in the probability of success compared with teeth with unacceptable quality (*P =* 0.006, OR = 0.135; 95% CI, 0.032–0.563). In contrast, there was no statistically significant difference between success rate and family income, father’s and mother’s education level, healthcare provider’s educational level, microscope use, treatment system used, obturation technique, quality of RC filling, presence of normal lamina dura at treatment time, presence of normal periodontal ligament space at treatment time, and history of the presence of periapical lesions (Table 3).

**Table 3:** Binary Logistic Regression Analysis showing Factors Affecting Success Rate Based on Loose Criteria.

Variable	Successful Based on Loose Criteria			*P*-value	OR (95% CI)
	Yes Mean/n (%)	No Mean/n (%)	Total n (%)		
*Sociodemographic characteristics*					
Patient’s age at treatment time	14.4	13.2	-	0.036∗	0.981 (0.963–0.999)
Family Income					
Low	19 (8.4)	15 (25.9)	34 (11.9)	0.372	0.316 (0.025–3.966)
Moderate	195 (86.3)	31 (53.4)	226 (79.6)	0.301	0.380 (0.061–2.375)
Father’s Education Level					
Low	9 (4)	6 (10.3)	15 (5.3)	0.388	0.403 (0.051–3.169)
Moderate	39 (17.3)	20 (34.5)	59 (20.8)	0.287	0.447 (0.102–1.968)
Mother’s Education Level					
Low	63 (27.9)	27 (46.6)	90 (31.7)	0.086	4.459 (0.809–24.570)
Moderate	78 (34.5)	16 (27.6)	94 (33.1)	0.477	1.709 (0.390–7.486)
*Treatment characteristics*					
Time Elapsed between Treatment Time and Assessment Time					
6–12 months	42 (18.6)	16 (27.6)	58 (20.4)	0.111	3.501 (0.749–16.368)
13–24 months	33 (14.6)	11 (19)	44 (15.5)	0.043∗	4.558 (1.049–19.806)
25–36 months	37 (16.4)	13 (22.4)	50 (17.6)	0.191	2.756 (0.602–12.606)
37–48 months	31 (13.7)	10 (17.2)	41 (14.4)	0.179	3.080 (0.597–15.896)
Healthcare Provider Training Level					
Undergraduate students	2 (0.9)	11 (19)	13 (4.6)	0.216	8.359 (0.290–240.725)
Interns	19 (8.4)	12 (20.7)	31 (10.9)	0.931	0.876 (0.044–17.370)
General dentist	4 (1.8)	3 (5.2)	7 (2.5)	0.577	0.354 (0.009–13.553)
Post-graduate students	190 (84.1)	12 (20.7)	202 (71.1)	0.814	0.716 (0.044–11.590)
Consultant	6 (2.7)	19 (32.8)	25 (8.8)	0.195	6.357 (0.389–103.97)
Microscope Use					
Used	172 (76.1)	6 (10.3)	178 (62.7)	0.142	0.243 (0.037–1.605)
Not used	24 (10.6)	19 (32.8)	43 (15.1)	0.615	1.515 (0.300–7.668)
Treatment System					
Conventional	11 (4.9)	17 (29.3)	28 (9.9)	0.572	1.730 (0.259–11.544)
Rotary	190 (84.1)	16 (27.6)	206 (72.5)	0.615	0.675 (0.146–3.122)
Obturation Technique					
Lateral	24 (10.6)	18 (31)	42 (14.8)	0.673	0.659 (0.095–4.586)
Vertical	7 (3.1)	1 (1.7)	8 (2.8)	0.780	0.616 (0.021–18.286)
*Dental characteristics*					
Radiographic quality of coronal restoration at treatment time (Acceptable)	221 (97.8)	36 (62.1)	257 (90.5)	0.025∗	0.170 (0.036–0.799)
Radiographic quality of RC filling at treatment time (Acceptable)	205 (90.7)	32 (55.2)	237 (83.5)	0.255	0.487 (0.141–1.682)
LD at treatment time (Normal)	116 (51.3)	15 (25.9)	131 (46.1)	0.998	1.001 (0.250–4.005)
PDL at treatment time (Normal)	149 (65.9)	15 (25.9)	164 (57.7)	0.485	0.581 (0.126–2.669)
Presence of PA at treatment time (Present)	176 (77.9)	35 (60.3)	211 (74.3)	0.818	1.142 (0.369–3.540)

LD: Lamina Dura.

PDL: Periodontal ligament.

PA: Periapical.

∗ Statistical significance at P-value ≤0.05.

***Based on strict criteria,*** binary logistic regression analysis was conducted, which revealed a statistically significant (*P* ≤ 0.05) relationship between success and time lapse between treatment and assessment time, the use of a microscope, and radiographic quality of coronal restoration at treatment time. A shorter time lapse between treatment and assessment resulted in a statistically significant lower probability of success compared to a longer time lapse (*P* = 0.001, OR = 8.703, 95% CI:2.357 to 32.130; *P* = 0.019, OR = 4.689, 95% CI:1.291 to 17.031). Moreover, the use of a microscope resulted in a statistically significant increase in the probability of success (*P =* 0.038, OR = 0.183; 95% CI, 0.037–0.912). In addition, teeth with acceptable restoration quality had a statistically significant increased probability of success compared to teeth with unacceptable quality (*P =* 0.005, OR = 0.044; 95% CI, 0.032–0.563). On the other hand, there was no statistically significant difference between the prevalence of success and family income, education level of the father and mother, educational level of healthcare provider, treatment system used, obturation technique, quality of RC filling, presence of normal lamina dura at treatment time, presence of normal periodontal ligament space at treatment time, or history of presence of periapical lesion (Table 4). Therefore, the null hypothesis is rejected.

**Table 4:** Binary Logistic Regression Analysis showing Factors Affecting Success Rate Based on Strict Criteria.

Variable	Successful Based on Strict Criteria			*P*-value	OR (95% CI)
	Yes Mean/n (%)	No Mean/n (%)	Total n (%)		
*Sociodemographic characteristics*					
Patient’s age at treatment time	14.5	13.5	-	0.078	0.987 (0.973–1.001)
Family Income					
Low	15 (7.2)	19 (24.7)	34 (12)	0.777	1.373 (0.153–12.357)
Moderate	180 (87)	46 (59.7)	226 (79.6)	0.930	1.075 (0.212–5.447)
Father’s Education Level					
Low	6 (2.9)	9 (11.7)	15 (5.3)	0.788	1.304 (0.188–9.025)
Moderate	35 (16.9)	24 (31.2)	59 (20.8)	0.481	0.630 (0.174–2.280)
Mother’s Education Level					
Low	58 (28)	32 (41.6)	90 (31.7)	0.673	1.318 (0.365–4.753)
Moderate	71 (34.3)	23 (29.9)	94 (33.1)	0.565	0.723 (0.240–2.178)
*Treatment characteristics*					
Time Elapsed between Treatment Time and Assessment Time					
3–12 months	34 (16.4)	24 (31.2)	58 (20.4)	0.001∗	8.703 (2.357–32.130)
13–24 months	31 (15)	13 (16.9)	44 (15.5)	0.091	3.108 (0.835–11.564)
25–36 months	31 (15)	19 (24.7)	50 (17.6)	0.019∗	4.689 (1.291–17.031)
37–48 months	31 (15)	10 (13)	41 (14.4)	0.331	2.033 (0.486–8.512)
Healthcare Provider Training Level					
Undergraduate students	1 (0.5)	12 (15.6)	13 (4.6)	0.537	2.956 (0.094–92.486)
Interns	16 (7.7)	15 (19.5)	31 (10.9)	0.187	0.160 (0.010–2.437)
General dentist	4 (1.9)	3 (3.9)	7 (2.5)	0.090	0.045 (0.001–1.629)
Post-graduate students	176 (85)	26 (33.8)	202 (71.1)	0.365	0.324 (0.028–3.708)
Consultant	6 (2.9)	19 (24.7)	25 (8.8)	0.902	1.172 (0.095–14.458)
Microscope Use					
Used	164 (79.2)	14 (18.2)	178 (62.7)	0.039∗	0.161 (0.028–0.910)
Not used	18 (8.7)	25 (32.5)	43 (15.1)	0.335	2.294 (0.424–12.408)
Treatment System					
Conventional	8 (3.9)	20 (26)	28 (9.9)	0.316	2.756 (0.379–20.021)
Rotary	177 (85.5)	29 (37.7)	206 (72.5)	0.726	1.319 (0.280–6.209)
Obturation Technique					
Lateral	19 (9.2)	23 (29.9)	42 (14.8)	0.770	0.768 (0.131–4.500)
Vertical	7 (3.4)	1 (1.3)	8 (2.8)	0.704	0.571 (0.032–10.300)
Dental characteristics					
Radiographic quality of coronal restoration at treatment time (Acceptable)	204 (98.6)	53 (68.8)	257 (90.5)	0.008∗	0.044 (0.004–0.451)
Radiographic quality of RC filling at treatment time (Acceptable)	191 (92.3)	46 (59.7)	237 (83.5)	0.269	0.511 (0.155–1.683)
LD at treatment time (Normal)	111 (53.6)	20 (26)	131 (46.1)	0.141	0.453 (0.158–1.301)
PDL at treatment time (Normal)	140 (67.6)	24 (31.2)	164 (57.7)	0.495	1.540 (0.446–5.317)
Presence of PA at treatment time (Present)	164 (79.2)	47 (61)	221 (74.3)	0.548	0.745 (0.285–1.944)

LD: Lamina Dura.

PDL: Periodontal ligament.

PA: Periapical.

∗ Statistical significance at P-value ≤0.05.

## 4 Discussion

This study aimed to evaluate the success and failure rates of RCT in first permanent molars (FPMs) among children. The first permanent molar was deemed crucial for occlusion. Angle hypothesized that “the first permanent molar, more than any other tooth or anatomical point, provides a precise scientific basis for defining occlusal disharmony and occlusal anomalies”. [18]. The first permanent molar is the first permanent tooth to erupt in the oral cavity distal to the second primary molar [19] at the age of 6–7 years [20]. They are the teeth most susceptible to environmental conditions such as caries, hypoplasia, or hypomineralization.

In the current study, clinical and radiographic examinations were performed for RC-treated FPMs to determine the success and failure rates of treatment. The total number of teeth assessed in this study was 284. Most of the evaluated teeth 226/284 (79.6%) had successful treatments based on loose criteria. While 207/284 out of (72.9%) teeth had successful treatment based on strict criteria. However, only of 58/284 (20.4%) teeth were considered to have failed.

Among this study sample, more than half of the included sample (61.3%) of patients were females. This is in line with the results of Ng et al*.* [21] in their study, who reported that 58% of RCT were conducted for female patients. In this study, mandibular FPMs were most frequently treated. This might be due to the fact that mandibular FPMs have higher caries rate than their maxillary counterpart. This explanation was reported by Mimoza and Vito [22], who found that FPMs located in the mandible have a higher caries prevalence than maxillary FPMs. Moreover, Chen et al. [23] emphasized this finding by reporting higher caries prevalence among mandibular molars than among maxillary molars.

In this study, 226 teeth were deemed successful based on loose criteria, representing a success rate of 79.6%. Similarly, Fonzar et al. [24] reported that out of 1175 teeth, 1034 (88%) had successful treatments, 988 (84.1%) teeth were considered a complete success, and partial success was reported in 46 (3.9%) teeth. Contrary, in the literature, higher level of treatments survival was reported by Benenati and Khajotia in their study [25]. They combined the percentage of successful n = 555 (62.08%) and acceptable n = 259 (28.97%) cases, presenting an overall percentage of 91.05% [25]. Another study reported the cumulative 4 years survival rate following RCT to be 95.4% [21].

In the current study, patients with a mean age of 14.4 years had an increased probability of success compared with younger patients. This could be due to the fact that performing RCT in pediatric patients compared to adults is considered challenging, while the canals of the teeth might still be wide as tertiary dentin is not yet formed. Treated teeth may have structural defects, such as hypoplasia or hypomineralization. Several other factors could affect treatment prognosis, including patient cooperation, pain tolerance in pediatric patients, and the feasibility of providing proper coronal restorations following treatment.

In the present study, a shorter time lapse between treatment and assessment resulted in a lower probability of success compared to a longer time lapse. This is in line with the findings of Salehrabi and Rotstein [8] in their work, who evaluated the outcomes of RCT among a large population of patients in the United States of America (USA) and reported that the unfortunate outcomes of RC-treated molar teeth occurred in the first three years following treatment.

In this study population, the success rate of RCT based on strict criteria was 72.9%. This result is in line with the results of the study conducted by Heling and Tamshe [26]. They reported the success rate among their study sample, which included 213 RC-treated teeth, to be 70% [26]. In the study performed by Benenati and Khajotia [25] to evaluate the success rate of nonsurgical RCT done by undergraduate students at the University of Oklahoma, College of Dentistry, USA, and reported that among 894 RC-treated teeth, the overall success rate was 62.08% [25]. Fonzar et al. [24] reported that complete success was found in 988 (84.1 %) of their sample of teeth (n = 1175). Morse et al. [27] in their study performed a radiographic analysis to determine the success rate of 220 teeth. They found an overall success rate of 94.5%. Moreover, Sjogren et al*.* [28] reported that the overall success rate of RCT in their study sample, consisting of 635 teeth, was 91% after 8–10 years.

In the present study, it was found that teeth with acceptable coronal restoration quality had significantly higher success based on loose and strict criteria. The effect of coronal restoration on RCT success has been assessed in previous studies [17, 29]. In their systematic review and meta-analysis, Gillen et al. [17] reported that adequate coronal restorations together with adequate root filling increased the odds for apical periodontitis healing. A previous study demonstrated that the success rate of adequately filled RC-treated teeth with adequate coronal restoration was 91.4%, while the success rate of adequately filled RC-treated teeth with inadequate coronal restoration was found to drop to 44% [29]. This result is consistent with the conclusion of Tavares et al., who reported that coronal restorations can affect the RCT outcomes. In other words, coronal restorations of acceptable quality were found to significantly decrease the prevalence of apical periodontitis significantly [17].

This study has several limitations. The study was conducted in three centers in one city. However, Jeddah City is considered the second largest city in the Kingdom of Saudi Arabia and the eighth largest city in the Middle East It is a widely heterogeneous population [30]. Therefore, conducting research in this geographic location would be valuable for both researchers and the community. The centers included in the selected city covered a wide geographic area and treated patients from different backgrounds. In addition, the data of the study sample were retrieved from the Information Technology (IT) department responsible for the electronic filing system from 2010 to 2019, which is considered a long period, and several confounding factors could affect the treatment outcome. Furthermore, a post-hoc analysis was conducted to assess whether there was enough sample power to support the study hypothesis. An odd-ratio of “2”, a R^2^ other X of 0.1 and α error probability of 0.05 resulted in 99% power.

Another limitation is that the sample was convenient. However, the distribution of the sample was assisted by sex, and both excluded and included subjects showed a similar prevalence. A future prospective cohort study design that includes different healthcare centers to control for confounding factors is recommended.

## 5 Conclusion

Among the studied samples, the success rate of RCT was found to be high among children. Performing RCT among older aged children, using the microscope and providing coronal restoration with acceptable quality would increase the probability of success.

## 6 Clinical significance

Determining the success and failure rates of root canal treatment in first permanent molars among children, as well as the factors affecting the outcome, would help in making appropriate clinical decisions.

## Declarations

### Author contribution statement

Wala Dhafar; Nada Bamashmous; Heba Sabbagh: Conceived and designed experiments; performed the experiments; Analyzed and interpreted data; Contributed reagents, materials, analysis tools or data; Wrote the paper.

Abdullah Albassam; Sanaa Alhamed: Conceived and designed experiments; Wrote the paper.

Jihan Turkistani; Rzan Zaatari; Manal Almalik; Ahlam Bahkali: Contributed reagents, materials, analysis tools or data.

Amal Dafar: Analyzed and interpreted data.

### Funding statement

This research did not receive any specific grant from funding agencies in the public, commercial, or not-for-profit sectors.

### Data availability statement

Data will be made available on request.

### Declaration of interest’s statement

The authors declare no conflict of interest.

### Additional information

No additional information is available for this paper.
